# The Senegalese and Brazilian strains of *Schistosoma mansoni* exhibit high compatibility with *Biomphalaria pfeifferi* and *Biomphalaria glabrata*

**DOI:** 10.1186/s13071-025-06918-5

**Published:** 2025-07-31

**Authors:** Mbéré Sarr, Malick Diop, Abdoulaye Jacque Bakhoum, Souleymane Doucoure, Doudou Sow, Cheikh Tidiane Bâ, Cheikh Sokhna, Jérôme Boissier, Bruno Senghor

**Affiliations:** 1 MINES, UCAD-IRD International Campus, Hann, 18524 Dakar, Senegal; 2 Department of Animal Biology, Cheikh Anta Diop University of Dakar, Dakar, Senegal; 3 Department of Parasitology-Mycology, Health Sciences Training & Research Unit, University Gaston Berger, 234 Saint-Louis, Senegal; 4 Aix Marseille University, IRD, AP-HM, SSA, RITMES, 27 Boulevard Jean Moulin, 13005 Marseille, France; 5 Mediterranean Infection University Hospital Institute, 19-21 Boulevard Jean Moulin, 13005 Marseille, France; 6 Host-Pathogen-Environment Interactions (IHPE) Laboratory, CNRS, IFREMER, University of Montpellier, University of Perpignan Via Domitia, Perpignan, France; 7 Immuno-Physiophathology and Infectious Diseases Unit, Pasteur Institute of Dakar, 36 Pasteur Avenue, Dakar, Senegal

**Keywords:** *Schistosoma mansoni*, *Biomphalaria glabrata*, *Biomphalaria pfeifferi*, Senegal, Brazil, Compatibility

## Abstract

**Background:**

Intestinal schistosomiasis, caused by *Schistosoma mansoni*, is endemic in both Africa and South America. In Senegal and Brazil, *S. mansoni* is transmitted by *Biomphalaria pfeifferi* and *Biomphalaria glabrata*, respectively. With increasing human migration from Senegal to the Americas, there is a potential risk of transferring parasite strains across continents. Understanding the compatibility between *Schistosoma* species and strains, and snail hosts is therefore essential. This study investigated the compatibility of two *S. mansoni* strains from Senegal (SmSEN) and Brazil (SmBRA) with both *B. pfeifferi* (BpSEN) and *B. glabrata* (BgBRA) originating from Senegal and Brazil, respectively.

**Methods:**

Four infection combinations were performed: (1) SmSEN + BpSEN (2) SmSEN + BgBRA (3) SmBRA + BgBRA, and (4) SmBRA + BpSEN. A minimum of 72 snails were individually exposed to five miracidia per combination.

**Results:**

The data show high compatibility between Brazilian and Senegalese *S. mansoni* with *B. pfeifferi* (92.4% and 77.3%, respectively). In contrast, both strains showed a low compatibility with *B. glabrata*, with rates of 67.3 and 48% for the Brazilian and Senegalese *S. mansoni*, respectively.

**Conclusions:**

The high compatibility between *S. mansoni* and *Biomphalaria* species from Senegal and Brazil highlights the adaptability of *S. mansoni* to infect different *Biomphalaria* species across geographically distinct regions. These findings emphasize the importance of monitoring areas at risk of schistosomiasis emergence, particularly in the context of human migration and the invasive spread of *Biomphalaria* species into novel environments.

**Graphical abstract:**

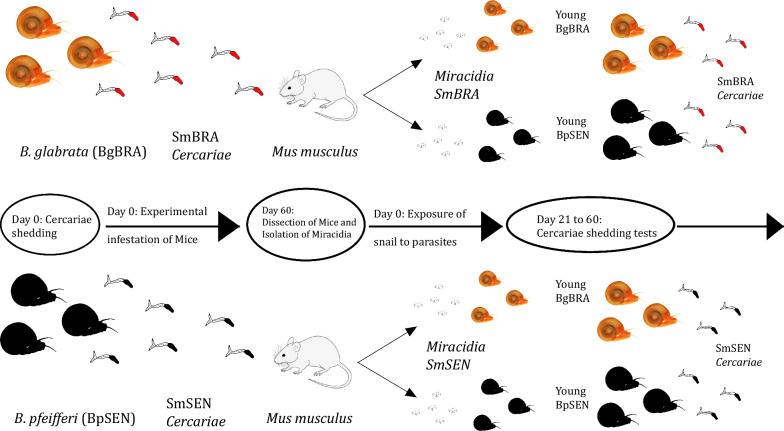

## Background

Human schistosomiasis is a neglected tropical disease caused by trematodes of the genus *Schistosoma*. In terms of health and socioeconomic impact, it ranks as the second most common parasitic disease after malaria and is the most widespread waterborne disease in tropical and subtropical regions [1]. Globally, schistosomiasis remains a major public health concern, with approximately 780 million people at risk, 240 million infected, and an estimated 280,000 deaths annually [1, 2].

Six *Schistosoma* species are known to infect humans. Among them, *Schistosoma haematobium*, *S. mansoni*, and *S. japonicum* are the most common, while *S. intercalatum*, *S. guineensis*, and *S. mekongi* are less frequently encountered [3]. Sub-Saharan Africa bears the highest disease burden, accounting for over 90% of cases, mainly due to *S. haematobium* and *S. mansoni* [4].

*S. mansoni*, which causes intestinal schistosomiasis, is widely distributed across intertropical Africa, South America (particularly Brazil), and the Caribbean [5]. The majority of individuals infected with *S. mansoni* live in sub-Saharan Africa, where approximately 54 million are infected and 393 million are at risk [6]. In Brazil, *S. mansoni* remains a significant public health issue, affecting six million people and placing 25 million at risk [7].

The transmission of *S. mansoni* depends on freshwater snails of the genus *Biomphalaria*, which serve as intermediate hosts. Two species are particularly important in West Africa and South America: *Biomphalaria pfeifferi* and *Biomphalaria glabrata* [8]. In Senegal, *B. pfeifferi* is prevalent in the Senegal River Valley in the north [9] and the Kédougou region in the south [10, 11]. In the Americas, *B. glabrata* is a primary snail intermediate host for *S. mansoni* [8, 12]*.*

For more than 50 years, researchers have studied the compatibility between laboratory or natural strains of *S. mansoni* and *Biomphalaria* from sympatric (the same geographic origin) and allopatric (different origin) foci. These studies have revealed significant polymorphism in compatibility, depending on the specific *S. mansoni* strains and *Biomphalaria* species tested [13–18]. Several factors influence the compatibility status of snails and schistosomes, including, geographical origin of both snails and parasites [13, 17, 19–21], immunological interactions [14, 16, 22], snail size and age at exposure, and number of miracidia used in infections [17, 19]. These findings underscore the complexity of host–parasite compatibility [23] depicting where *Schistosoma* species and strains are endemic or can be introduced too.

Phylogenetic evidence supports a close genetic relationship between *S. mansoni* populations in West Africa and the Americas, suggesting that African strains were introduced to the New World via the transatlantic slave trade [24–27]. However, the precise African origins of these introduced strains remain uncertain. In recent years, human migration from Africa to Latin America has grown steadily [28]. Many African migrants, particularly Senegalese, transit through multiple Latin American countries en route to North America or settle in South American countries such as Argentina and Brazil [29–32]. This migration trend raises concerns about the importation of the Senegalese *S. mansoni* strains into parts of South America, particularly in areas with poor sanitation and frequent water contact, impacting local transmission.

In this context, the present study aims to evaluate the compatibility of Brazilian and Senegalese *S. mansoni* strains with both *B. glabrata* and *B. pfeifferi*.

## Method

### Strain origins

All *Schistosoma* and *Biomphalaria* cultures and all experiments were performed at the Neglected and Emerging Diseases in the South Unit (MINES) laboratory in Senegal.

*Biomphalaria* spp.: The albino Brazilian strain of *Biomphalaria glabrata* (BgBRA) originated from Recife in Brazil (1975) was obtained from the Host–Pathogen–Environment Interactions (IHPE) laboratory in Perpignan, France. The *Biomphalaria pfeifferi* (BpSEN) strain was a wild population collected from the Lampsar stream (16° 06′ 33.3″ N–16° 20′ 58.2″ W) of the Senegal River basin in June 2022. Snail populations were maintained separately in aquaria filled with dechlorinated water, equipped with air pumps for aeration. The snails were regularly fed fresh lettuce leaves. Healthy juvenile BgBRA and BpSEN snails were used as experimental intermediate hosts.

*S. mansoni strains*: *S. mansoni* from Brazil (SmBRA) was isolated in 1975 from Recife (Brazil) and was supplied to the IHPE by Professor Y. Golvan from the Sorbonne University Faculty of Medicine, St Antoine, Paris (France) before being established at MINES [31]. The Senegalese *S. mansoni* strain (SmSEN) was obtained from naturally infected *B. pfeifferi* snails collected from the Lampsar stream of the Senegal River basin in 2022.

### Establishing the SmSEN and SmBRA rodent infections

BpSEN snail’s natural shedding *S. mansoni* cercariae were used for the SmSEN rodent infections whereas laboratory BgBRA snails experimentally infected with the SmBRA strain were used for the SmBRA rodent infections. Five infected snails of each species (BpSEN/SmSEN and BgBRA/SmBRA) were placed in separate glass pillboxes containing 5 ml of spring water. The pillboxes were exposed to a 40-W neon lamp for 30 min to stimulate the release of the *S. mansoni* cercariae. Cercariae of each strain, SmSEN and SmBRA, were collected and transferred to 10 prelabeled plastic containers (five for each strain), containing spring water 1–2 cm deep. These containers contained ~150 cercariae and were maintained at a controlled temperature of 25 °C, in preparation for the subsequent exposure of the mice.

### Mice exposure

Laboratory animal experiments followed the standard methods established by the IHPE [32, 33]. Female mice (*Mus musculus*) of 45 days old were individually exposed to SmSEN (*n* = 5) or SmBRA (*n* = 5) cercariae. Prior to exposure, each mouse was placed in a container filled with 3 cm of clean water to allow for acclimatization. The mice were exposed to the cercariae for 45 min–1 h. The animals were then transferred to labeled cages and were kept in their groups of five.

### Snail infections

At 60 days postexposure, the mice from each group were euthanized using a combination of Rompun and Imalgene at a dosage of 0.1 ml per 10 g of body weight, followed by an intraperitoneal injection of sodium pentobarbital (100 mg/kg, 0.1 ml). After euthanasia, the livers were removed from the carcass, homogenized in saline solution, and passed through a series of sieves with progressively smaller mesh sizes (360 µm, 180 µm, 106 µm, 75 µm, and 40 µm). The *S. mansoni* eggs were collected in the final sieve. The livers of each group of five mice (SmSEN or SmBRA) were processed together.

The eggs were washed into Petri dishes with distilled water and exposed to a 40-W neon lamp for 15–30 min to stimulate miracidia hatching. Using a micro pipette, five miracidia were collected under a dissecting microscope and deposited into individual wells of a 24-well plastic microplate. The number of miracidia in each well was checked, and adjustments were made as necessary. Juvenile BgBRA and BpSEN, aged 1–2 weeks and measuring 2–4 mm in size, were placed individually into each well containing the five miracidia. Four infection combinations were performed: (1) SmSEN + BpSEN (2) SmSEN + BgBRA (3) SmBRA + BgBRA and (4) SmBRA + BpSEN. A minimum of 72 snails were individually exposed to five miracidia per combination. Following exposure, the microplates were covered overnight with a black cloth to prevent the snails from escaping.

### Snail maintenance

Snails were maintained in the MINES laboratory in Senegal using established IHPE protocols [32, 33] and adapted to the local laboratory conditions in Senegal. At 24 h postexposure, the snails were transferred to clearly labeled snail tanks. A maximum of 24 snails of each infection combination were placed in each tank measuring 35 cm long, 23.5 cm wide, and 8.5 cm high. The tanks were covered by a translucent polypropylene cover with several small holes for breathing. The snails were kept at ambient temperature between 22 °C and 26 °C with a natural light–dark cycle. For the first 2 weeks, the snails were fed with dried salad leaves and subsequently with a combination of fresh and dried leaves. Dechlorinated tap water, aged for 2 weeks at room temperature [34], was used for each tank and changed at 3–5 day intervals. The snail mortality rate for each combination was recorded daily until day 60 postexposure.

### Determining infections

The prepatent periods of the *S. mansoni* strains were monitored beginning on day 21 postexposure. From day 21 to day 60 postexposure, snails were monitored daily for cercarial shedding. They were individually placed in wells of 24 well-plates containing spring water and exposed to artificial light. Cercariae emergence was observed using a dissecting microscope. The numbers of snails that were observed to shed for each infection combination were recorded.

### Statistical analysis

For each infection combination, data were recorded on the number of surviving snails, the number exposed, and the number that became positive. The infection rate for each species was calculated as the ratio of the number of positive snails to the number exposed. To compare mortality rates and infection success across the combinations, Fisher’s exact test was used https://www.socscistatistics.com/-tests/fisher/default2.aspx. A *P*-value of less than 0.05 was considered statistically significant.

## Results

### Prepatent period

Differences in the prepatent period were observed. The SmSEN/BgBRA combination had a prepatent period of 23 days, whereas all other combinations, SmSEN/BpSEN, SmBRA/BpSEN, and SmBRA/BgBRA, had a prepatent period of 25 days (Table 1).

**Table 1:** Infection data for *Biomphalaria glabrata* and *Biomphalaria pfeifferi* exposed to *S. mansoni* from Senegal and Brazil

Parasite	Origin of* S. mansoni*			
Country	Senegal (SmSEN)		Brazil (SmBRA)	
Snail species	*B. glabrata* (BgBRA)	*B. pfeifferi* (BpSEN)	*B. glabrata* (BgBRA)	*B. pfeifferi* (BpSEN)
Number exposed	112	96	110	72
Number died	46	38	9	11
% (95% CI)	41.07% (31.96–50.18)	39.58 (29.80–49.37)	8.18% (3.06–13.30)	15.28% (7.88–25.69)
*P*-value	0.8876		0.1415	
Prepatent period	25	25	25	23
Number tested	66	58	101	66
Number positive	32	45	68	61
Rate of infection (95% CI)	48.48% (36.43–60.54)	77.59% (66.85–88.32)	67.33% (58.18–76.47)	92.42% (86.04–98.81)
*P*-value	0.0009		0.0001	

### Mortality rate

Mortality rates were significant (*P* < 0.00001) higher for both snail strains (BpSEN 41% and BgBRA 39%) when exposed to the Senegalese wild *S. mansoni* (SmSEN) strain compared with snails (BpSEN 8% and BgBRA 15%) exposed to the Brazilian laboratory *S. mansoni* (SmBRA) strain. However, there were no significant differences in snail mortality rates when exposed to the same *S. mansoni* wild or laboratory strain (*P* = 0.8876 and 0.1415, respectively) (Table 1).

### Compatibility of Senegal *S. mansoni* (SmSEN) with Brazil *B. glabrata* (BgBRA) and Senegal *B. pfeifferi* (BpSEN)

Of the 66 BgBRA and 58 BpSEN snails exposed to the SmSEN strain, 32 (48.5%) and 45 (77.6%) snails were successfully infected and shed cercariae, respectively. Infectivity rates were significantly higher for the SmSEN/BpSEN combination compared with the SmSEN/BgBRA combination (*P* = 0.0009) (Table 1).

### Compatibility of *Brazil S. mansoni* (SmBRA) with *B. glabrata* (BgBRA) and *B. pfeifferi* (BpSEN)

Of the 101 BgBRA and 66 BpSEN snails exposed to the SmBRA strain, 68 (67.32%) and 61 (92.4%) were successfully infected and shed cercariae, respectively. Infectivity rates were significantly higher in the SmBRA/BpSEN combination compared with the SmBRA/BgBRA combination (*P* < 0.00001) (Table 1).

## Discussion

This study assessed the compatibility of wild *B. pfeifferi* from Senegal (BpSEN) and laboratory *B. glabrata* from Brazil (BgBRA) with a field-derived *S. mansoni* strain from Senegal (SmSEN) and laboratory-adapted *S. mansoni* strain originally from Recife, Brazil (SmBRA, NMRI strain). Notable differences in key life-history traits, including prepatent periods, snail mortality, and infection rates were observed for all the different *S. mansoni*/*Biomphalaria* combinations, highlighting the variability in snail–schistosome interactions.

Little variation (2 days) was observed for the prepatent periods for all combinations. The shortest prepatent period, 23 days, occurred with the SmBRA/BpSEN combination, while the longest period (25 days) was recorded for both the SmSEN/BpSEN and SmSEN/BgBRA combinations. These results are broadly consistent with earlier studies that reported prepatent periods that varied by a 1–2 days between different *S. mansoni/Biomphalaria* combinations: 21 days for Senegalese *B. pfeifferi*/Cameroonian *S. mansoni* [40], 19–25 days *S. mansoni* from Kinshasa (Congo) infecting *Biomphalaria camerunensis*, and 25 days *S. mansoni* and *B. alexandrina* from Egypt [41]. Our results also disputed the theory that *Schistosoma*-snail pairings involving species that are coendemic exhibit shorter prepatent periods compared with those that are not, owing to differences in immune responses [42].

Our data show a significantly higher mortality rate for *B. glabrata* and *B. pfeifferi* exposed to the Senegalese *S. mansoni* strain that was isolated from natural populations compared with those exposed to the lab-adapted Brazilian *S. mansoni* strain. This pattern aligns with previous findings, where *B. glabrata* from Brazil had 87% mortality when exposed to wild Cameroonian *S. mansoni* versus 51% mortality when infected with a laboratory *S. mansoni* strain from Guadeloupe [42]. These discrepancies are likely attributed to the strong selection pressures that occur in maintaining laboratory *Schistosoma* and snail strains. However, our study did not include unexposed control groups, which limits our ability to definitively attribute mortality to parasite infection rather than other factors.

Our data show higher compatibility between *S. mansoni* and *B. pfeifferi*, irrespective of the origin compared with *B. glabrata* for both the SmSEN and SmBRA strains. These findings are consistent with earlier studies demonstrating the high susceptibility of *B. pfeifferi* with *S. mansoni* [41, 43–45] compared with *Biomphalaria sudanica* [46, 47].

Interestingly, the SmBRA strain had a very high infection rate of 92.42% with BpSEN, compared with 67.33% rate with its sympatric host snail BgBRA. Portet et al. (2019) also reported high infection rates using the same *S. mansoni* strain and Senegalese *B. pfeifferi* [41], suggesting a strong affinity between *S. mansoni* from South America and *B. pfeifferi* from Senegal. However, it is acknowledged that this *S. mansoni* strain (known as Naval Medical Research Institute (NMRI) strain) is a well adapted laboratory strain and so may not reflect the natural *S. mansoni* population. In contrast, higher compatibility was observed between Senegalese *B. pfeifferi* and *S. mansoni* (SmSEN/BpSEN) that are sympatric, supporting the hypothesis that *Schistosoma* strains are locally adapted to their native snail species/strains [15, 44, 45, 48, 49].

Even though the infection rate of BgBRA with SmSEN was only 48%, this still supports the theory that African *S. mansoni* strains successfully colonized the Americas via the transatlantic slave trade (approximately 400–500 years ago) [24–27]. The SmSEN strain was not molecularly characterized to confirm species identity. However, this limitation is mitigated by the fact that the only other *Schistosoma* species known to infect *Biomphalaria* in Africa is *Schistosoma rodhaini*, which is only reported to occur in East Africa (namely Rwanda, Uganda, and Tanzania) [35–39], making its presence in Senegal highly unlikely.

Genetic studies suggest a Central African origin for *S. mansoni* in South America, with no direct evidence implicating *S. mansoni* from West African countries [25]. However, there is clearly a potential for Senegalese *S. mansoni* to infect *B. glabrata* in South America, highlighting that *Schistosoma* species and strains can be introduced to new areas, via migration, with poor sanitation and contact with freshwater containing susceptible host snails.

The potential for African *Schistosoma* species to infect local snail populations in Europe was illustrated by the recent outbreak of urogenital schistosomiasis in Corsica, facilitated by the presence of *Bulinus truncatus*, established human migration routes with West Africa and urinary egg excretion (high risk of contamination of natural swimming pools) [50]. This highlights the need to monitor areas where schistosomiasis could be introduced via human migration, owing to the invasiveness of *Biomphalaria* species into novel areas [8].

## Conclusions

The high level of compatibility between *S. mansoni* and *Biomphalaria* species from Senegal and Brazil highlight the adaptability of *S. mansoni* to infect different *Biomphalaria* species across geographically distinct regions. While the results support hypotheses regarding historical parasite–host associations, particularly the introduction of African *S. mansoni* into South America, such interpretations remain speculative without further evidence. To better assess the risk of parasite dissemination and clarify the historical dynamics of schistosomiasis transmission, future studies should include wild-caught Brazilian snails and genetically characterized *S. mansoni* strains from both continents.

## Data Availability

Data supporting the main conclusions of this study are included in the manuscript.

## Abbreviations

BgBRA: *Biomphalaria glabrata* originating from Brazil
BpSEN: *Biomphalaria pfeifferi* originating from Senegal
MINES: Infectious, Neglected and Emerging Diseases in the South Unit
SmBRA: *Schistosoma mansoni* originating from Brazil
SmSEN: *Schistosoma mansoni* originating from Senegal
